# Visualization of asymmetric wetting ridges on soft solids with X-ray microscopy

**DOI:** 10.1038/ncomms5369

**Published:** 2014-07-10

**Authors:** Su Ji Park, Byung Mook Weon, Ji San Lee, Junho Lee, Jinkyung Kim, Jung Ho Je

**Affiliations:** 1 X-ray Imaging Center, Department of Materials Science and Engineering, Pohang University of Science and Technology, San 31, Hyoja-dong, Pohang 790-784, South Korea; 2 School of Advanced Materials Science and Engineering, SKKU Advanced Institute of Nanotechnology (SAINT), Sungkyunkwan University, Suwon 440-746, South Korea

## Abstract

One of the most questionable issues in wetting is the force balance that includes the vertical component of liquid surface tension. On soft solids, the vertical component leads to a microscopic protrusion of the contact line, that is, a ‘wetting ridge’. The wetting principle determining the tip geometry of the ridge is at the heart of the issues over the past half century. Here we reveal a universal wetting principle from the ridge tips directly visualized with high spatio-temporal resolution of X-ray microscopy. We find that the cusp of the ridge is bent with an asymmetric tip, whose geometry is invariant during ridge growth or by surface softness. This singular asymmetry is deduced by linking the macroscopic and microscopic contact angles to Young and Neuman laws, respectively. Our finding shows that this dual-scale approach would be contributable to a general framework in elastowetting, and give hints to issues in cell-substrate interaction and elasto-capillary problems.

## Abstract (web summary)

Wetting on soft surfaces exhibits a ridge structure at the contact line and the underlying mechanism is not yet fully understood. Here, Park *et al.* visualize the evolution of the ridge tip with high spatial and temporal resolution and identity its asymmetric shape independent of surface softness.

## Graphical abstract

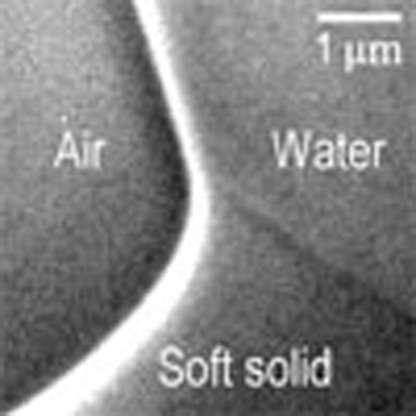

Many natural and synthetic materials are soft and deformable owing to the nature as in rubbers or to their structure as for thin films^1^,^2^,^3^ and fibres^4^,^5^,^6^. Wetting behaviour of soft deformable solids, that is, elastowetting, is a very old subject and much attention has been paid over the past half century^1^,^2^,^3^,^4^,^5^,^6^,^7^,^8^,^9^,^10^,^11^,^12^,^13^,^14^,^15^,^16^,^17^,^18^,^19^,^20^,^21^,^22^,^23^,^24^,^25^,^26^ for various scientific issues involved in soft tissues^27^,^28^,^29^,^30^,^31^,^32^ and polymer gels^33^, as well as for practical applications such as inkjet printing^34^ and microfluidic devices^35^. Recently, the interaction of the contact line with a deformable substrate, which causes a local microscopic deformation, that is, the formation of a ‘wetting ridge’, has been actively investigated^7^,^8^,^9^,^10^,^11^,^12^,^13^,^14^,^15^,^16^,^17^,^18^,^19^,^20^,^21^,^22^,^23^,^24^,^25^ since the pioneering work on the first measurement of the ridge by Carré *et al.*^20^

Wetting behaviours on solids have been explained by Young law (cos*θ*_L_=(*γ*_SV_–*γ*_SL_)/*γ*_LV_)^36^, based on a force balance of interfacial tensions (*γ*_LV_, *γ*_SV_ and *γ*_SL_ of liquid–vapour, solid–vapour and solid–liquid interfaces, respectively) that determines the equilibrium contact angles (*θ*_S_, *θ*_L_ and *θ*_V_ in solid, liquid and vapour, respectively) at the three-phase contact line.

On a soft solid, however, the vertical component of liquid surface tension, which is not considered in Young law, has critical roles in the wetting behaviours, such as the wrinkling^1^,^2^ or folding^3^ of thin films and the bending of fibres^4^,^5^,^6^. The vertical component, which is highly concentrated at the microscopic region of the three-phase contact line, induces a local microscopic structure on the soft surface, that is, a ‘wetting ridge’. Despite many theoretical^7^,^8^,^9^,^10^,^11^,^12^,^13^,^14^,^15^,^16^ and experimental^7^,^17^,^18^,^19^,^20^,^21^,^22^,^23^,^24^,^25^ studies over the past half century, the microscopic geometry of a wetting ridge, particularly, its ‘tip geometry’, still remains unresolved.

The first measurement of the wetting ridge was achieved by using white-light interferometry, which was limited to the dry side of the ridge far from the tip^20^. Since then, many researchers have tried to find the tip using optical techniques such as confocal microscopy^7^,^18^,^19^ or optical profilometry^21^. However, direct observation of the tip for precise measurements of the contact angles is hardly achievable by optical imaging owing to limited resolutions^7^,^18^,^19^,^20^. For instance, confocal microscopy^18^ failed to directly visualize the vicinity of the contact line due to strong light scattering over the detection limit by the steep slopes near the contact line. Jerison *et al.*^7^ first measured triangular cusps by embedding fluorescence particles on the surface in confocal microscopy. The resolution of this approach, however, was not high enough to visualize the tip and precisely measure the geometry of the cusp. Here we present the first, direct and real-time visualization of ridge tips with high spatial and temporal resolutions using X-ray microscopy, and discuss a general framework of wetting on soft solids based on precise measurement of the geometry of the tips.

## Results

### Direct visualization of ridge tips

**Figure 1:**
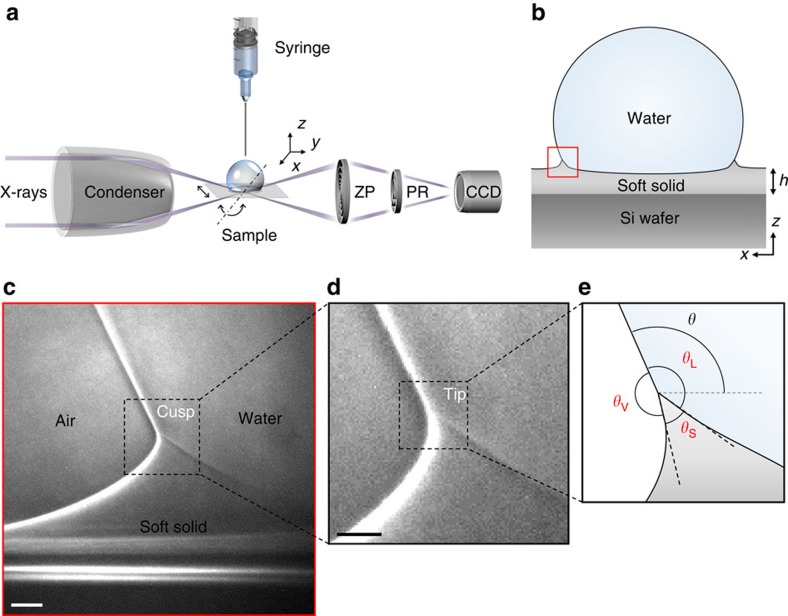
High-resolution X-ray imaging for wetting ridge formation. (**a**,**b**) Schematic illustrations of (**a**) transmission X-ray microscopy (TXM) and (**b**) a sessile drop on a soft substrate. TXM consists of a capillary condenser, a motorized sample stage, a zone plate (ZP), a phase ring (PR) and a CCD camera. A wetting ridge (red square in **b**) is formed by the surface tension of a water drop at the contact line. (**c**,**d**) Demonstration of a directly visualized (**c**) wetting ridge with (**d**) a bent cusp (magnified image of the dashed square in **c**) and an asymmetric tip (dashed square in **d**). (**e**) Extraction of three interfaces from the ridge tip (magnified image of the dashed square in **d**), which enables us to measure the macroscopic (*θ*) and the microscopic (*θ*_S_, *θ*_V_ and *θ*_L_) contact angles. (**c**,**d**) Scale bars, 2 and 1 μm, respectively.

For this study, we used transmission X-ray microscopy (TXM), as illustrated in Fig. 1a, to provide direct visualization of a wetting ridge for a water (or 40% ethylene glycol aqua solution (EG 40%)) drop (*r*≈1 mm) on a silicone gel (or polydimethylsiloxane (PDMS)) surface (Fig. 1b). All of the three interfaces, particularly including the LV interface, at the tip of a wetting ridge were clearly visualized, as demonstrated in Fig. 1c, with a high spatial resolution (~50 nm per pixel) without using any contrast agents. The interference bright and dark fringes at each interface, originating from the Zernike phase contrast^37^,^38^, allowed us to clearly identify each interface^39^,^40^. We for the first time found that wetting ridges in large drops (*r*≈1 mm) have bent cusps (Fig. 1d; dashed square of Fig. 1c) with the asymmetric tips rotated (Fig. 1e; dashed square of Fig. 1d) towards the LV interfaces (Fig. 1d,e). The rotation of the tips observed here is different from the reported rotation of the whole ridges that is due to the substantial dimple generated under small droplets (*r*<<250 μm) by large Laplace pressures^19^. High-resolved images of the tips of cusps enabled us to accurately measure the microscopic (*θ*_S_, *θ*_L_ and *θ*_V_) as well as the macroscopic (*θ*) contact angles (Fig. 1e; dashed square of Fig. 1d).

### Surface profiles

**Figure 2:**
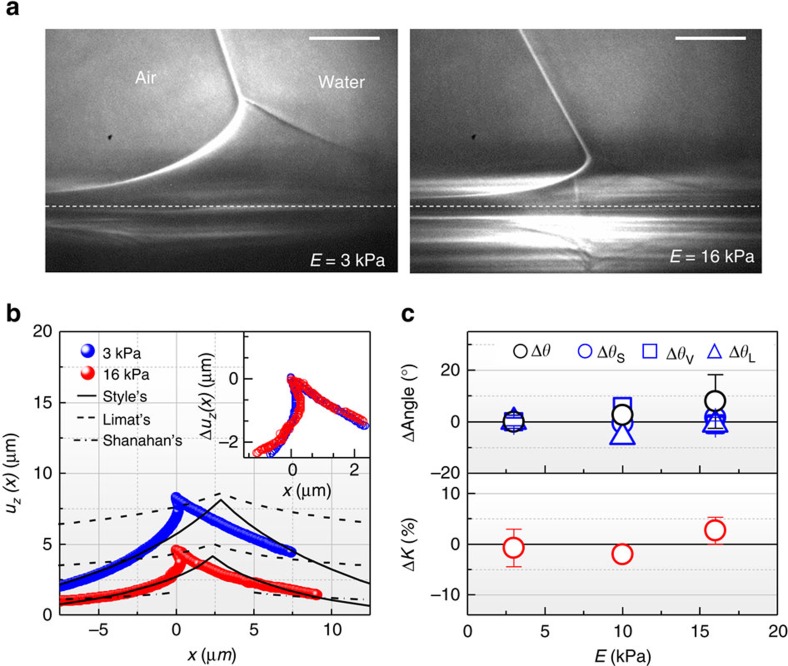
Effect of surface elasticity on wetting ridge formation. (**a**) Representative X-ray images of wetting ridges on a silicone gel (*E*≈3 kPa) and a PDMS film (≈16 kPa). Scale bars, 5 μm. (**b**) The surface profiles clearly show a strong *E*-dependence of the vertical displacement *u*_*z*_(*x*). The asymmetric and bent cusps are compared with three LE symmetrical models by Style *et al.*^8^ (solid lines), Limat^12^ (dashed lines) and de Gennes and Shanahan^10^,^11^,^20^ (dash-dotted lines). For *E*≈3 kPa, the model by Shanahan is invalid in the observed region here. The detailed fitting descriptions are in Supplementary Fig. 2. (Inset) The cusps are identically superimposed by Δ*u*_*z*_(*x*)=*u*_*z*_(*x*)−*u*_*z*_(0) at *w*<<*l*_e_. (**c**) (Top) The angular differences (ΔAngle) are plotted with *E*, based on the average values for 3 kPa, for the macroscopic (*θ*, black circle) and the microscopic (*θ*_S_ (blue circle), *θ*_V_ (blue square) and *θ*_L_ (blue triangle)) angles measured at *w*< ~0.4 μm. The error bars are s.d. from five sets of image data for 3 kPa, one set for 10 kPa and three sets for 16 kPa. (Bottom) *K* is obtained with *θ*_S_ and *γ*_LV_, that is, *K*=sin*θ*_S_/*γ*_LV_. The little difference of *K* (Δ*K*<4%), resulted from that of Δ*θ*_S_ (< ±1.6°), indicates invariant surface stresses *Υ*_SL_ and *Υ*_SV_ at the tips.

The direct visualization made it possible to accurately extract the surface profiles *u*_*z*_(*x*) (vertical surface displacement at *x*) of wetting ridges (Supplementary Fig. 1), as demonstrated for two surfaces with *E* (elasticity)=3 and 16 kPa in Fig. 2a,b. The two profiles (blue and red circles in Fig. 2b) clearly show asymmetric and bent cusps. Here the horizontal peak position of each surface profile was set as *x*=0. We tried to fit the following three linear elastic (LE) symmetrical models to these profiles (Supplementary Fig. 2): (i) the model by de Gennes and Shanahan^10^,^11^,^20^ (dash-dotted lines) based on the classical stress analysis of an elastic film by a concentrated normal force; (ii) the model by Limat^12^ (dashed lines) based on symmetric surface energies (*γ*_SV_=*γ*_SL_=*γ*_S_, that is, *θ*=90°); and (iii) the model by Style and Dufresne^7^,^8^ (solid lines) based on symmetric surface stresses (*Υ*_SL_=*Υ*_SV_=*Υ*_S_), where *Υ*_SL_ (*Υ*_SV_) is the surface stress of the SL (SV) interface. The best fits of the three models are displayed in Fig. 2b.

The surface profiles predicted by most of LE symmetrical models^7^,^8^,^9^,^10^,^11^,^12^,^13^,^14^,^15^,^20^ are not well matched with our experimental data (Fig. 2b and Supplementary Fig. 2), except the model by Style and Dufresne^8^ in EG 40% (Supplementary Fig. 2f). In fact, the predicted profiles themselves are quite different from each other. The heights of ridges predicted ~*γ*_LV_sin*θ*/*E* are relatively in a good agreement with those measured in this study *u*_z_(0), though (Table 1).

The model by Shanahan and co-workers^10^,^11^,^20^ is inapplicable to predict the profiles in the cusp regions (*w* (ridge width) << *l*_e_ (=*Υ*_S_/*E*, the elasto-capillary length)). Actually, the cusp regions are inelastic regions (|*x−x′*|≲*ε*, where *x′* is the position of the contact line and *ε* is the cutoff distance^10^), based on the fact that the estimated *ε* are comparable to *l*_e_ for our systems (Table 1). Of course, the model is relatively well matched in the elastic zone (|*x−x′*|≳*ε*) the dash-dotted line (the green solid line) in |*x*|>~5 μm in Fig. 2b (Supplementary Fig. 2a), consistent with Fig. 2 of ref. 20.

The surface profiles by Limat^12^, who included symmetric surface energies (*γ*_SV_=*γ*_SL_=*γ*_S_) in the Shanahan’s description of surface displacement^10^,^11^,^20^, are furthermore far from our experimental data (Supplementary Fig. 2c,d; the solid lines are from surface energies, *γ*_S_=(*γ*_SV_+*γ*_SL_)/2 and the dashed lines are from surface stresses, *Υ*_S_=(*Υ*_SV_+*Υ*_SL_)/2). The deviation is presumably due to oversimplification, in particular, by the assumptions of *θ*_SV(SL)_ <<1 and *γ*_LV_ <<*γ*_S_, which are in contrast to our case (*θ*_SV_ >>1 and *γ*_LV_ (*Υ*_LV_)>*γ*_S_ (*Υ*_S_)), where *θ*_SV(SL)_ is the slope of the SV (SL) interface.

The best fits were obtained by using the model by Style and Dufresne^8^ (Fig. 2b and Supplementary Fig. 2e,f) that accounts for the surface tension of solid, that is, the surface stress. For the water system with asymmetric surface energies (*γ*_SV_≠*γ*_SL_), the surface profiles are relatively in a good agreement with our data in the region of *w*>>*l*_e_, but not in the region of *w*<<*l*_e_, where the cusp shows significant asymmetry (Fig. 2b and Supplementary Fig. 2e). For the EG 40% system with symmetric surface energies (*γ*_SV_≈*γ*_SL_), the profiles (in particular, the green solid line) in Supplementary Fig. 2f correspond to the experimental data in overall region. However, this symmetrical model intrinsically fails to capture the microscopic contact angles at ridge tips that are actually asymmetric even in symmetrical surface energies.

The profiles, especially, in the region where *w*<<*l*_e_, are significantly deviated from the best fits of the three models, presumably due to large asymmetry and/or large strain at the tips. In calculating *u*_*z*_(*x*) in Fig. 2b, we assumed *Υ*_S_=(*Υ*_SL_+*Υ*_SV_)/2 or *γ*_S_=(*γ*_SV_+*γ*_SL_)/2, where *Υ*_SL_ (*Υ*_SV_) was measured from our experimental data and *γ*_SV_ (*γ*_SL_) was extracted from literatures^41^,^42^. Over the region where *w*>>*l*_e_, however, the model by Style and Dufresne^8^ is better matched to our experimental data than the other models^10^,^11^,^12^,^20^. We note that in any systems with *γ*_SV_≈*γ*_SL_ (that is, *θ*≈90°) or a small *γ*_LV_, the ridge profiles can be approximately symmetric, except their tips (Supplementary Fig. 2f). In fact, Style and Dufresne^7^,^19^ observed symmetric triangular cusps in the large drop limit (*r*>>250 μm), which were very close to their model despite the simple approximation. However, their approach is in principle not correct as it neglects any source of asymmetry, in particular, the difference between *Υ*_SL_ and *Υ*_SV_. The accurate observation of the tip geometry has been unattainable so far.

### Solid elasticity

More interestingly, we found that the shapes of two cusps in our experimental data are nearly identical or self-similar (the inset of Fig. 2b), regardless of *E* or the ridge height *u*_*z*_(0) (~*γ*_LV_sin*θ* /*E*)^7^,^8^,^10^,^12^,^14^,^20^, which is consistent with other references^7^,^8^,^19^ except the asymmetry. To confirm the self-similarity, we measured the macroscopic and the microscopic contact angles (Fig. 1e) in a length of ~0.4 μm for various surface elasticities, as listed in Supplementary Table 1, and the angular dependencies on surface elasticity were plotted in Fig. 2c (top) based on the averaged values for *E*≈3 kPa. Interestingly, we found that the variation of each angle by elasticity is very small despite the strong elasticity dependence of *u*_*z*_(*x*). In particular, the small difference in Δ*θ*_S_ (< ±1.6°) leads to a nearly constant *K* (=sin*θ*_S_/*γ*_LV_) with *E* as in Fig. 2c (bottom, Δ*K* <4%), directly indicating that the two surface stresses of *Υ*_SL_ and *Υ*_SV_ are invariant with *E*, as deduced from Neuman relation (*K*=sin*θ*_S_/*γ*_LV_=sin*θ*_V_/*Υ*_SL_=sin*θ*_L_/*Υ*_SV_)^19^. Herein, surface stresses^43^ (specifically, *Υ*_SV_ and *Υ*_SL_), instead of surface energies (*γ*_SV_ and *γ*_SL_), are applied as effective interfacial tensions for SV and SL interfaces^19^, which also helps to overcome a paradox: a force balance at the tip but the violation of the Neumann triangle condition in terms of the surface energies as *γ*_W(or EG 40%)_>*γ*_PDMS_+*γ*_W(or EG 40%)–PDMS_ (Table 2). Here, we note that *θ*_S_ is in good agreement with the calculated value from the asymmetric case of Limat’s model^12^ (Supplementary Table 2 and Supplementary Note 1), which indicates that asymmetry of surface stresses or surface energies is undoubtedly a critical factor determining the ridge tip geometry.

A rather large deviation in the macroscopic angle (*θ*) presumably results from the evaporation of the water drops and the heterogeneity of the soft surfaces. We found that the macroscopic angle (*θ*=108.1±9.0°) for the soft surfaces is consistent to that (*θ*=106.6±2.3°) for rigid surfaces (*E*≈750 kPa; see Methods section). This suggests that Young law would hold regardless of *E*. We note that herein Young law is valid in terms of surface energies in macroscopic scale (drop size: approximately millimetre), where the contribution of the local deformation (approximately micrometre) at the contact line to the surface stresses is negligible. Here the effect of the Laplace pressure is negligible for large droplets (*r*>>250 μm) as in our case (*r*≈1 mm; Table 1 and Supplementary Note 1), different from the large Laplace pressure for small droplets (*r*<<250 μm) that causes significant rotation of the ridge and decrease of the ridge height by making a large dimple under the droplets^19^.

### Ridge-growth dynamics

**Figure 3:**
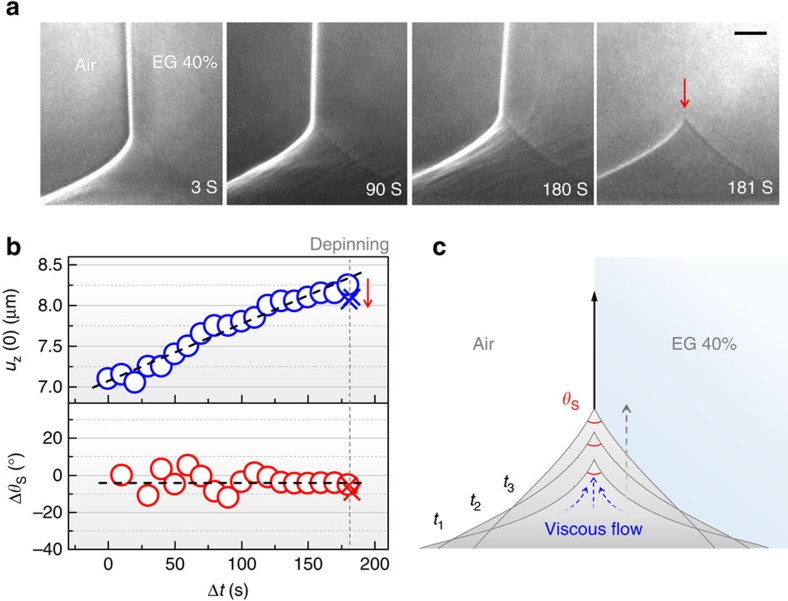
Ridge-growth dynamics and its effect on cusp formation. (**a**) Representative sequential snapshots of a cusp during ridge growth for a EG 40% drop on a silicone gel (*E*≈3 kPa and *h*≈50 μm; Supplementary Movie 1). The cusp was instantly recovered (red arrow) right after depinning at 181 s (grey dotted line). Scale bar, 2 μm. (**b**) (Top) The ridge height *u*_*z*_(0) increases at a constant rate of ~7 nm s^−1^ until depinning at Δ*t* (observing time)=181 s. The abrupt decrease right after depinning (red arrow) is attributed to an instantaneous elastic recovery. (Bottom) Δ*θ*_S_ is unchanged during the ridge growth (*θ*_S_=56.3±5.1°). X-shaped symbols in **b** is the values obtained after depinning. Black dashed lines are a guide to the eye. (**c**) A schematic illustration of ridge growth from *t*=*t*_1_ to *t*=*t*_3_. Invariant *θ*_S_ during the slow and linear ridge growth in **b** might be caused by a liquid-like viscous flow in the soft substrate.

To explore wetting dynamics on soft solids, ridge-growth dynamics should be first studied but is rarely known with few observations^22^,^23^. We used non-volatile EG 40% drops, instead of evaporating water drops, during dynamic experiment. High temporal resolution of TXM enabled us to take real-time movies of ridges during their growth. Figure 3a demonstrates a ridge growth for a EG 40% drop on silicone gel (*E*≈3 kPa), recorded 1–2 min after contact line pinning until the contact line is depinned by injecting EG 40% into the drop (Supplementary Movie 1). We plotted both the ridge height *u*_*z*_(0) and the change of the solid contact angle Δ*θ*_S_ (within *w*< ~0.7 μm) as a function of observing time in Fig. 3b. We find that *u*_*z*_(0) linearly increases at a rate of~7 nm s^−1^. Here the abrupt decrease in *u*_*z*_(0) at 181 s (red arrows in Fig. 3a,b) is due to the depinning. Very interestingly, the contact angles (*θ*_S_) at all the ridge tips observed were nearly invariant despite their gradual increase in *u*_*z*_(0), as demonstrated in Fig. 3b.

## Discussion

**Figure 4:**
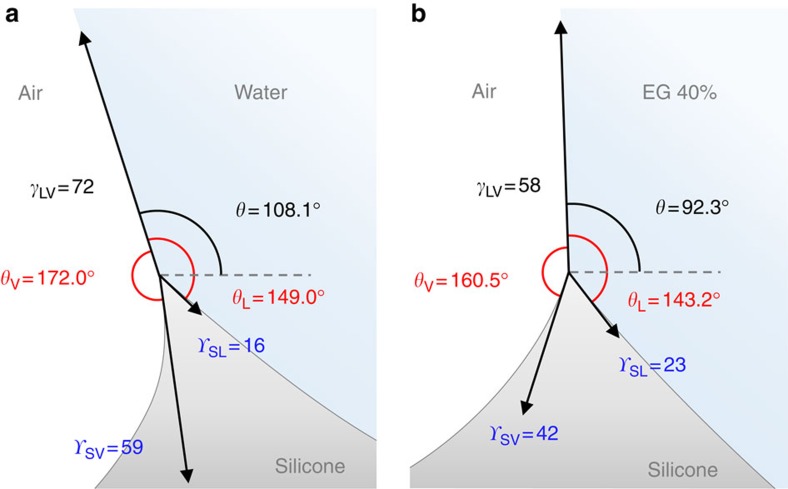
Microscopic force balances at the asymmetric and bent tips. (**a**,**b**) The estimated force balances at the asymmetric and bent tips for (**a**) water and (**b**) EG 40%, respectively. The liquid surface tension (*γ*_LV_) and the surface stresses (*Υ*_SL_ and *Υ*_SV_) are given in mN m^−1^.

Geometric invariance in the tip (*w*<<*l*_e_) during ridge growth or by surface softness indicates that cusp formation is controlled by capillarity and not by elasticity^7^,^8^. For a large drop with a negligible line tension and a negligible Laplace pressure, only three interfacial tensions can affect ridge formation. Herein, we exclude the possible existence of an extra tangential force by the long-range interaction between liquid and solid molecules suggested in refs 13, 14, as discussed in detail in Supplementary Fig. 3, Supplementary Table 3 and Supplementary Note 1. We illustrated the force balances at the ridge tips for water and EG 40% drops in Fig. 4. Here we see that the measured surface stresses are asymmetric (*Υ*_SV_≠*Υ*_SL_), regardless of whether *γ*_SV_≈*γ*_SL_ or *θ*≈90° (Fig. 4). This results in the formation of the asymmetric tips (*θ*_V_≠*θ*_L_). The difference in the surface stresses is because of their dependence on the mediums surrounding the surfaces^44^. The surface stresses can be also affected by surface elastic deformation that depends on the vertical component of the liquid surface tension. This explains the different surface stresses (*Υ*_SV(W)_>*Υ*_SV(EG 40%)_) in the two systems with different surface tensions (*γ*_W_>*γ*_EG 40%_) (Table 2). The precise measurement of the cusp geometry by our approach enabled us to find the asymmetric cusp geometries and thus to obtain the surface stresses accurately. The SV surface stress *Υ*_SV_ was measured as 59 (42) mN m^−1^ in a water (EG 40%) drop on silicone gel using our approach, which is larger than that (31 (28) mN m^−1^ in a glycerol (fluorinated oil) drop on silicone gel) measured by confocal microscopy^19^.

The bending of the cusp, clearly depicted in Fig. 4a, is attributed to the inclination of the LV interface with *θ*>90°, which is due to the asymmetric surface energies (*γ*_SV_≠*γ*_SL_). It is conceivable that by Neuman law the microscopic angles of the tip are established with respect to the inclined LV interface, leading to the rotation of the tip and thus the bending of the cusp (*w*<<*l*_e_). These results suggest that the geometry of the cusp with the rotated tip is determined by simultaneously applying macroscopic and microscopic force balances by Young and Neuman laws, respectively, as suggested by Style *et al.*^8^ More importantly, our results show applicability of such a dual-scale approach to a general framework of wetting on soft solids within a wide softness range and with asymmetric surface energies/stresses.

The time-invariant tip geometry (Fig. 3), which indicates time-invariant surface stresses, suggests that the ridge growth observed would be due to an inelastic deformation^43^. Specifically, the slow and linear growth of the ridges with the invariant contact angles (*θ*_S_) suggests that the ridge growth is, as illustrated in Fig. 3c, caused by a viscous flow in the soft solid, similar to that in a very viscous liquid^22^. Viscoelastic deformation of the ridge has been also observed even on elastomers, as reported by some irreversible part of the ridge growth for long residual times (approximately a few min) of a drop^24^. Further study is required to correlate the ridge growth and the plastic deformation.

In conclusion, we present the first direct visualization of wetting ridge tips on soft solids using high-resolution TXM. We revealed that the tip geometry of the wetting ridges has asymmetry and the cusps are bent by the inclined LV interfaces. From the measurements of the microscopic and macroscopic contact angles at their tips, we found that the microscopic geometry of the tip is invariant during the ridge growth or by the surface elasticity. The singular geometry of the cusps can be deduced by simultaneously applying Young and Neuman laws for macroscopic and microscopic force balances, respectively, as suggested by Style *el al*.^8^, but with using asymmetry of surface energies and surface stresses in our case. Our results show that the dual-scale approach would be applicable to general wetting behaviours on soft solids, beyond the limit of a linear elasticity or the assumption of symmetric surface energies/stresses^7^,^8^,^10^,^11^,^12^,^20^. Furthermore, our approach is a general technique that can be used to measure accurate surface stresses for soft materials within a wide stiffness range. This report would open a new subject in wetting on soft solids, especially regarding study on asymmetric ridge tip. Broadly speaking, X-ray microscopy would be helpful to investigate characteristic wetting behaviours on soft solids associated with spreading^20^,^21^,^22^, contact angle hysteresis^23^,^24^,^25^ or evaporation^23^,^25^, and to study various elasto-capillary phenomena^26^.

In this report, we suggest an applicability of a simple approach applying both Young and Neuman laws to dual-scale force balance and continuous surface deformation, which would be potentially important, particularly, in biological systems. For example, cell–substrate interactions might be closely linked to wetting behaviours on soft solids^17^; in fact, cells are known to sense and respond to the environment through small strains (3–4%) that are induced by their traction forces^28^. Indeed, the mechanical microenvironment is, as seen in cancer progression in breast epithelial cells^30^, critical to the normal development of most mammalian tissues and organs, which are soft viscoelastic materials (*E*=10^−1^ (for example, a brain) to 10^2^ kPa (for example, a cartilage))^27^. In addition, our approach might be practically applicable to estimating the cell traction force or the force-induced strain^29^, as well as the surface stresses^19^.

## Methods

### Materials and substrate fabrication

Pure water (Millipore) or EG 40% (Samchun Pure Chemical Co., Korea) drops of *r*≈1 mm were generated by a syringe pump and gently put on a silicone gel (*E*≈3 kPa; CY52-276A/B, Dow Corning Toray) or a PDMS (*E*≈10 or 16 kPa; Sylgard 184, Dow Corning Toray), spin coated with a controlled thickness (*h*=50 μm) on a Si wafer (Fig. 1b). Detailed protocols of preparing the substrates and their mechanical/rheological properties have been reported in refs 7, 19, 45, 46, 47. Si wafer as a solid support was used for the fine alignment of the samples to the X-ray beam, as confirmed by a mirror image of a wetting ridge (Fig. 2a) below the critical angle (0.22°) of Si.

### X-ray imaging

The experiment was conducted using TXM (Fig. 1a)^37^,^38^ at the 32-ID-C beamline in the Advanced Photon Source of the Argonne National Laboratory. We used a focused monochromatic X-ray beam at the photon energy of 9 keV (depth of focus: ~25 μm). The high brightness yielded high spatial resolution (50 nm per pixel) in a short acquisition time (50 ms per frame), much faster than reported^48^,^49^,^50^,^51^. A Fresnel zone plate was used as an objective lens. For phase-contrast enhancement, we used a Au Zernike phase ring, optimized for 9 keV, which is very useful to imaging soft matters such as organic^39^,^40^ or biological samples^37^,^38^ without any contrast agents. Image taking was started within 1–2 min after contact line pinning, under controlled temperature (22.5 °C) and humidity (19.5%).

### Data analysis

The contact angle *θ* for a rigid PDMS (*E*≈750 kPa) was, measured for five drops of water or EG 40% using optical microscopy, 106.6±2.3° or 95.8±0.7°, respectively. To test Young and Neuman conditions, we used the surface or interfacial tension of a liquid drop (PDMS oil or water (EG 40%)/PDMS oil) measured in the literatures^41^,^42^ (Table 2). Surface tension is the same as surface energy in liquid. Effects of X-ray irradiation^52^ to interfacial tensions or surface stresses are negligible, as confirmed by invariant contact angles. The surface profiles were extracted from the images using MATLAB and Image Pro-Plus 6.0 software (Supplementary Fig. 1)^51^,^52^. We calculated the surface profiles of the LE symmetrical models using MATLAB with the code for the model by Style *et al.*^8^ ( http://www.eng.yale.edu/softmatter/pubs.html). The surface profiles, calculated for different elastic moduli, were plotted in Supplementary Fig. 2.

## Author contributions

S.J.P., B.M.W. and J.H.J. designed the research; S.J.P., J.S.L., J.H.L. and J.K. performed the wetting experiments; S.J.P., B.M.W. and J.H.J. analysed the data; J.H.J. managed the project; S.J.P., B.M.W. and J.H.J. wrote the initial manuscript; and all authors discussed the results and commented for the final manuscript.

## Additional information

**How to cite this article:** Park, S. J. *et al.* Visualization of asymmetric wetting ridges on soft solids with X-ray microscopy. *Nat. Commun.* 5:4369 doi: 10.1038/ncomms5369 (2014).

## Supplementary Material

Supplementary Information Supplementary Figures 1-3, Supplementary Tables 1-3, Supplementary Note 1.

Supplementary Movie 1 Wetting ridge growth for an EG 40% drop on a silicone gel (E ≈ 3 kPa). The wetting ridge shows a slow and linear growth tendency. See Figure 3 and text.

## Figures and Tables

**Table 1:** Useful parameters.

Liquids (*E*)	*γ*_LV_sin*θ*/*E* (μm)	Exp. *u*_*z*_(0) (μm)	ε* (μm)	*l*_e_ (μm)	Δ*P*_L_ (kPa)	*F*(Δ*P*_L_)† (N m^−1^)
Water (3 kPa)	7.6	8.3	14.5	13	0.137	6.3E−3
Water (16 kPa)	1.4	4.6	2.7	2.5	0.137	1.7E−3
EG 40% (3 kPa)	6.4	7.4	12.3	10	0.116	4.7E−3

*E,* elasticity; EG 40%, 40% ethylene glycol aqua solution; *u*_*z*_, vertical surface displacement; *γ*_LV_, interfacial tension of liquid–vapour; *ε*, cutoff distance.

Starting from the left side of the table, the order of ridge height, the measured ridge height, the cutoff length scale, the elasto-capillary length scale, the Laplace pressure and the Laplace pressure term in equation (12) of ref. 11.

^*^ε were calculated from equation (5) in ref. 10.

^†^
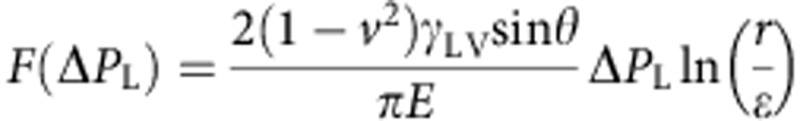
 from equation (12) in ref. 11.

**Table 2:** Interfacial tensions of liquids and estimated surface stresses.

Liquids	*γ*_LV_	*γ*_SV_	*γ*_SL_	*Υ*_SV_	*Υ*_SL_
Water	72	21*	40*	59	16
EG 40%	58	21*	28†	42	23

EG 40%, 40% ethylene glycol aqua solution; *γ*_LV_, *γ*_SV_ and *γ*_SL_ are interfacial tensions (liquid–vapour (LV), solid–vapour (SV) and solid–liquid (SL) interfaces, respectively); *Υ*_SV_ and *Υ*_SL_ are the surface stresses of solid vapour and solid–liquid, respectively.

The properties were obtained from the literature^41^,^42^.

Each value is given in mN m^−1^.

^*^Ref. 4141.

^†^Ref. 4242.
