# Supplementary material for: Visualization of asymmetric wetting ridges on soft solids with X-ray microscopy
Source: Nat Commun. 2014 Jul 10;5:4369. doi: 10.1038/ncomms5369 (PMC4104447; doi:10.1038/ncomms5369)
Supplement: Supplementary Information — Supplementary Figures 1-3, Supplementary Tables 1-3, Supplementary Note 1. [file ncomms5369-s1.pdf]

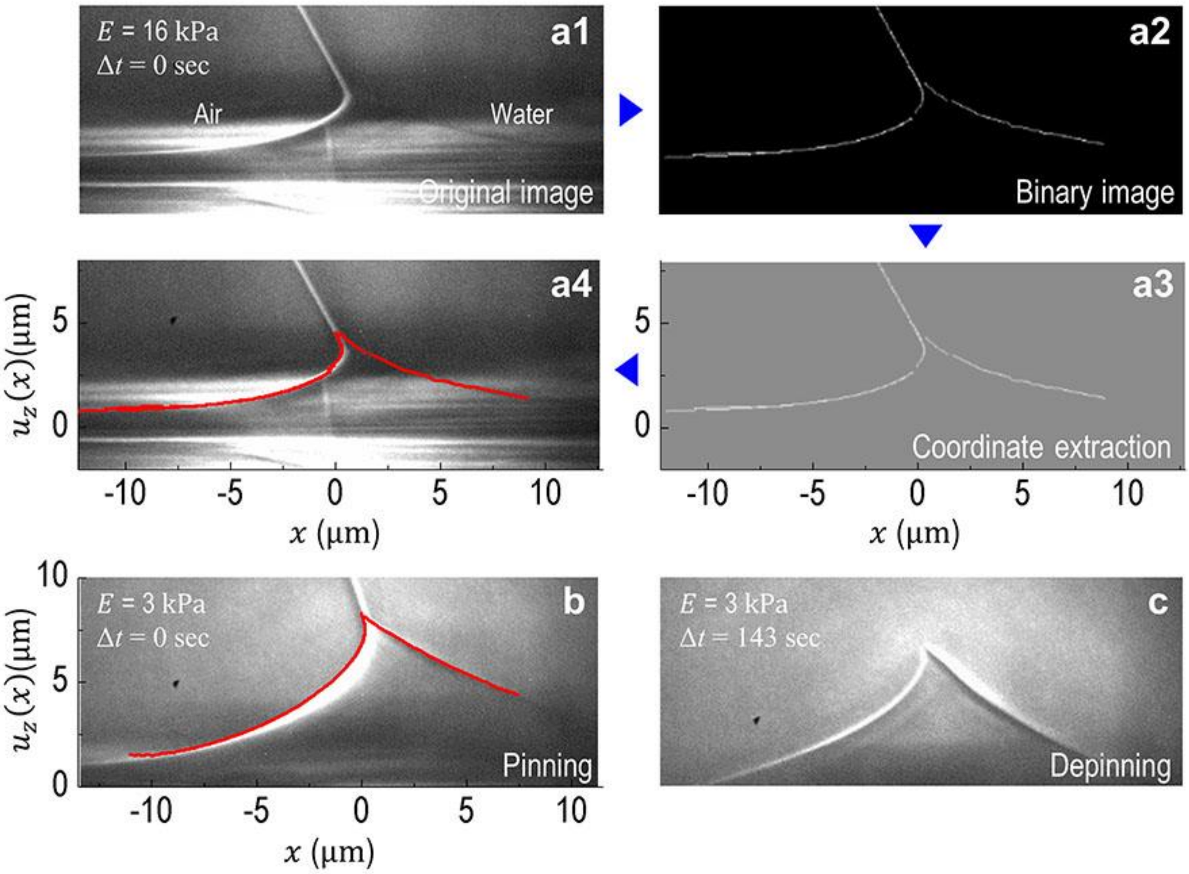

Supplementary Figure 1 | Extracting process of wetting ridge profiles. a1-4, An extraction example of a ridge profile for $E \approx 16$ kPa. An original image (**a1**) was binarized, as shown in **a2**, by Canny edge detector in MATLAB. The coordinates of the white pixels in the binary image (**a2**) were then extracted, as in **a3**. The coordinates of the line points that could not be recognized by the Canny detector due to their weak contrasts were measured using a manual measurements tool in Image-Pro Plus 6.0 software. **a4,** The extracted ridge profile (red dots) is completely matched with the SL and SV interfaces in the original image (**a1**). **b,** The extracted profile of a ridge image for $E \approx 3$ kPa at $\Delta t = 0$ s. **c,** The ridge image obtained right after depinning the contact line in **b** ($\Delta t = 143$ s in this case). Compared with the tip profile in **b**, the clear tip image in **c** supports the validity of the extraction process. Here the interference bright and dark fringes at each interface in **a4** and **b** are originated from the Zernike phase contrast[39,40].

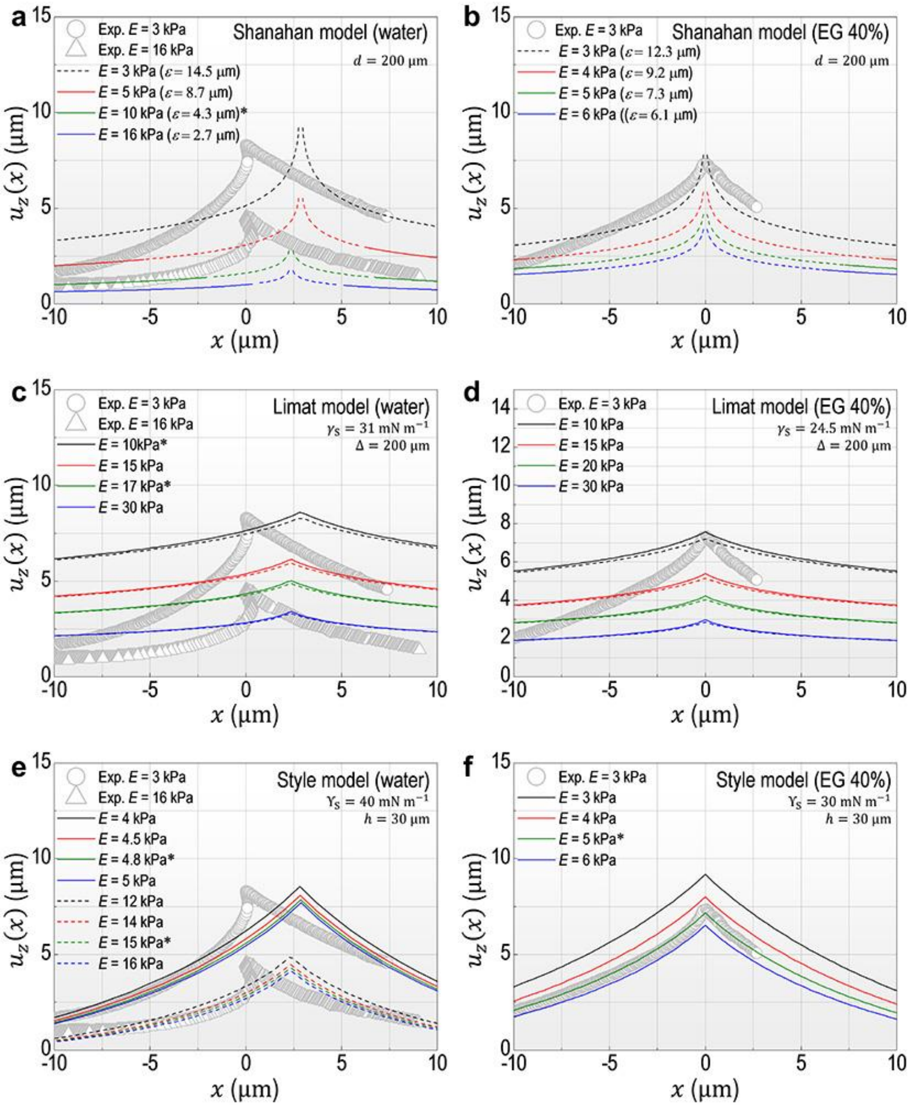

Supplementary Figure 2 | Surface profiles calculated from previous models. The models by (**a, b**) Shanahan *et al.*[10,11,20], (**c, d**) Limat[12], and (**e, f**) Style *et al.*[8] were calculated for (**a, c, e**) water and (**b, d, f**) EG 40% systems. Here, for asymmetric ridges in the water system, x' (the position of the contact line) for the calculated profiles were adjusted to the center of the ridges: $x' \approx 2.87$ µm (the center of Exp. E = 3 kPa) for the profiles of E = 3, 5 kPa in (**a**), 5, 10 kPa in (**c**), and 4~5 kPa in (**e**); $x' \approx 2.36$ µm (the center of Exp. E = 16 kPa) for the others. (**a, b**) For Shanahan's model, we used d = 200 µm, the distance from the contact line to unstrained part of the surface. The cut-off length ε was estimated for each elastic modulus E according to Eq. (5) in Ref. 10. We note that this model is invalid in the vicinity of the contact line, $|x - x'| < \varepsilon$ (dashed parts). (**c, d**) For Limat's model, we used the average values of the reported surface energies[50,51], $\gamma_S = (\gamma_{SV} + \gamma_{SL})/2$ (solid lines) or measured surface stresses, $\Upsilon_S = (\Upsilon_{SV} + \Upsilon_{SL})/2$ (dashed lines), and the macroscopic length scale Δ = 200 µm. (**e, f**) For the model by Style *et al.*, we used the average values of measured surface stresses, $\Upsilon_S = (\Upsilon_{SV} + \Upsilon_{SL})/2$ (see Fig. 4 and Table 2) and the film thickness h = 30 nm which is adjusted for the best fit. (*: the best fit in each model)

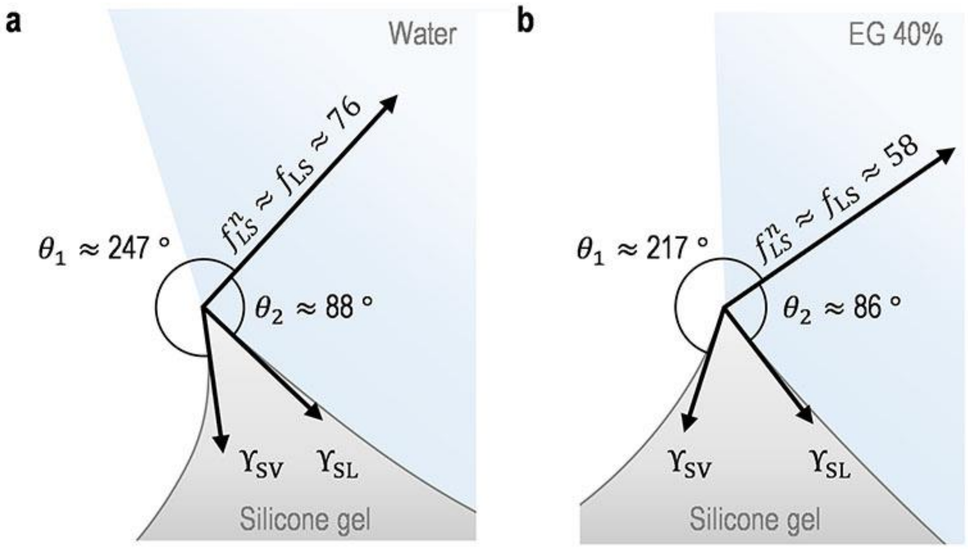

Supplementary Figure 3 | Schematic illustration of liquid on solid forces exerting on ridge-tips. a,b, The estimated liquid on solid forces at the asymmetric tips for (**a**) water and (**b**) EG 40% systems. The exact values of the surface stresses (Υ_{SL} and Υ_{SV}) are unknown. The angles between f_{LS} and SV interfaces are over 180°. All forces are given in mN m^{-1}.

Elasticity	No.	θ	θ_S	θ_V	θ_L
3 kPa	1	107.62	35.08	175.53	151.09
	2	102.53	39.07	171.88	150.99
	3	103.59	39.98	169.1	150.58
	4	108.77	37.7	168.43	153.8
	5	108.6	38.2	171.61	149.55
	Ave.	106.22	38.01	171.31	151.2
	SD	2.63	1.66	2.51	1.41
10 kPa	1	108.91	37.45	177.12	145.29
16 kPa	1	99.54	39.8	173.21	146.9
	2	119.38	41.02	170.86	148.31
	3	123.45	37.94	166.72	155.11
	Ave.	114.12	39.59	170.26	150.11
	SD	10.45	1.27	2.68	3.58

Supplementary Table 1 | Contact angles measured from x-ray images of wetting ridges on various soft substrates. The macroscopic (θ) and microscopic (θ_S, θ_V and θ_L) contact angles (°) were measured using Image-Pro Plus 6.0 software. For each elasticity condition, the average (Ave) and standard deviation (SD) were calculated.

Liquids	Symmetric case		Asymmetric case			Experimental data		
	θ_{SV} (°)	θ_{SL} (°)	θ_{SV} (°)	θ_{SL} (°)	θ_{S} (°)	θ_{SV} (°)	θ_{SL} (°)	θ_{S} (°)
Water	63.40	65.16	93.36	49.01	37.62	100.10	40.90	39.00
$\Delta\theta_{SX}$ (°)	–36.70	24.26	–6.74	8.11	–1.38			
EG 40%	66.84	68.69	79.06	59.29	41.65	72.80	50.90	56.30
$\Delta\theta_{SX}$ (°)	–5.96	17.79	6.26	8.39	–14.65			

Supplementary Table 2 | Comparison of the slope for each interface at the tip calculated from the Limat's model[12] to our experimental data. θ_{SV} (θ_{SL}) is the slope of the SV (SL) interface at a ridge-tip. In the calculation of a symmetric case ($\gamma_{SV} = \gamma_{SL}$), we used the average value of surface energies, $\gamma_S = (\gamma_{SV} + \gamma_{SL})/2$. θ_S is the microscopic angle of solid (see Fig 1e). Here, the angle difference $\Delta\theta_{SX} = \theta_{SX}$ (model) – θ_{SX} (exp.).

Liquids	$f_{\mathrm{LS}}^{t}/\gamma_{\mathrm{LV}}$	$f_{\mathrm{LS}}^{n}/\gamma_{\mathrm{LV}}$	f_{LS} (mN m^{-1})	$f_{z,\,\mathrm{norm}}$ (mN m^{-1})	$f_{z,\,\mathrm{vect}}$ (mN m^{-1})
Water	0.031	1.050	76	50	21
EG 40%	0.074	1.004	58	35	–3

Supplementary Table 3 | Calculation of the liquid on solid forces from the model in Ref. 13, 14.

Supplementary Note 1 | Equilibrium at the triple point

Surface energy. The validity of Neuman law has been discussed in the immediate proximity of the contact line ($w \lesssim 2\varepsilon$, i.e. the inelastic zone[10] or $w \lesssim t$ where t is the thickness of the liquid-vapor interface[9]). In our systems ($\gamma_{\mathrm{W(or\ EG\ 40\%)}} > \gamma_{\mathrm{PDMS}} + \gamma_{\mathrm{W(or\ EG\ 40\%)–PDMS}}$), the Neuman triangle condition is violated in terms of surface energies. Thus, we tried to check here other possible effects that have been proposed, as follows.

Laplace pressure. If we imagine a small drop with the Laplace pressure $\Delta P_{\mathrm{L}} = 2\gamma_{\mathrm{LV}}/r$, the solid surface near the contact line should undergo a typical rotation of order $\Delta\theta = \Delta P_{\mathrm{L}}/E$. In the limit of a contact angle, $\Delta P_{\mathrm{L}} \approx \gamma_{\mathrm{LV}}\theta/r$, which leads to a typical variation of local angles $\Delta\theta/\theta \approx l_{\mathrm{e}}/r$, where l_{e} is the elasto-capillary length. With r ~ 1 mm and l_{e} ~ 10 μm, as our systems, the variation $\Delta\theta/\theta \sim 10^{-2}$ is completely negligible. Although the angles of our systems are not small, the order of $\Delta\theta/\theta$ will not be changed. In addition to this scaling argument, we can also consider the ΔP_{L} term (= $(2(1-\nu^2)\gamma_{\mathrm{LV}}\sin\theta/\pi E)\Delta P_{\mathrm{L}}\ln(r/\varepsilon)$) in Shanahan's model[11]. In our systems ($E \sim 10^3$ Pa, $\gamma_{\mathrm{LV}} \sim 10^{-2}$ N m^{-1}, r ~ 1 mm, $\varepsilon \sim 10^{-6}$ m, and $\nu \sim 1/2$, where ν is the Poisson's ratio of the elastic material), the ΔP_{L} term is estimated as $\lesssim 10^{-3}$ N m^{-1} (see Table 1), which is small enough to be ignored compared with surface energies[11].

Disjoining pressure and direct elastic stress. White[15] suggested that the disjoining pressure and the direct elastic force in the three phase region play the role of a line tension. In his model, the disjoining pressure contribution ($\tau_{\Pi} \sim h_0\gamma_{\mathrm{LV}}$) and the elastic force contribution ($\tau_{\mathrm{E}} \sim \gamma_{\mathrm{LV}}y_0\sin\theta_{\mathrm{Y}}$) affect the deviation of the apparent macroscopic contact angle (θ) from the Young angle (θ_{Y}) by $\cos\theta = \cos\theta_{\mathrm{Y}} - (\tau_{\mathrm{E}} + \tau_{\Pi})/(\gamma_{\mathrm{LV}}r_{\mathrm{C}}) + O((h_0/r)^2)$ where h_0 is the vertical range of the disjoining pressure, r_{C} is the macroscopically apparent contact radius ($r_{\mathrm{C}} \approx r\sin\theta$), and y_0 is the vertical displacement of the substrate at the microscopic triple point ($\approx u_z(0)$). The disjoining pressure contribution estimated in our systems ($h_0 \approx 0.2$ nm for the van der Waals type disjoining pressure and $h_0/r \sim 10^{-6}$) is very small as ~ 10^{-11} N. The direct elastic contribution estimated $\tau_{\mathrm{E}} \sim 10^{-7}$ N is larger than τ_{Π}, but the deviation therefrom is very small as $\Delta\theta = \theta - \theta_{\mathrm{Y}} \sim 0.6$ °, which is in the range of experimental errors. These results indicate that the disjoining pressure contribution or the direct elastic contribution is ignorable in our analysis. In fact, the apparent contact angles measured in our systems (Supplementary Table 1) correspond to Young angles, i.e. $\theta \approx \theta_{\mathrm{Y}}$, regardless of E. This model also suggested that the microscopic angle of liquid be "0" in the region where $z << h_0$, which is, however, beyond our scope of resolution.

Liquid on solid force. We tested the normal force transmission model and the vectorial force transmission model by Snoeijer and Andreotti[13,14]. We calculated the liquid on solid force f_{LS}, which is the basis of the two models, for water or EG 40% (see Supplementary Table 3). In both models, the force balance at tips fails because the angle between f_{LS} and the solid-vapor interface is much larger than 180° in both water (~247°) and EG 40% (~217°) (see Supplementary Fig. 3). In fact, the tangential component f_{LS}^{t} (~$0.031\gamma_{\mathrm{LV}}$ and ~$0.074\gamma_{\mathrm{LV}}$ in water and EG 40%, respectively) is negligibly small compared with the normal component f_{LS}^{n} (~$1.050\gamma_{\mathrm{LV}}$ and ~$1.004\gamma_{\mathrm{LV}}$ in water and EG 40%, respectively), i.e. $f_{\mathrm{LS}} \approx f_{\mathrm{LS}}^{n}$ (see Supplementary Table 3). The inapplicability of the two models to our experimental data is presumably due to large asymmetry and/or large strain at the tips.

Symmetric surface stresses. Style & Dufresne[7,8] first adopted surface stress[37], $\Upsilon_{ij} = \gamma_{ij} + \delta\gamma_{ij}/\delta\varepsilon_e$, where ε_e is the elastic deformation, in their calculation of the surface deformation. In the limit of symmetrical surface energies ($\gamma_{SV} = \gamma_{SL}$) and surface stresses ($\Upsilon_{SL} = \Upsilon_{SV}$), their model gives a symmetrical cusp with slope $\pm\gamma_{LV}/\Upsilon_S$ either side, regardless of the elastic modulus E. The slope corresponds to the Neuman triangle after linearization for small surface gradients. The E-independency of slope is consistent with our experimental observation (Fig. 2). In addition, as showed in Supplementary Fig. 2f, the surface profile simulated using the surface stresses, which were estimated from our data based on the force balance among γ_{LV}, Υ_{SL}, and Υ_{SV}, is well matched to that measured for the symmetrical case (EG 40%).

Symmetric/asymmetric surface tensions. We also tested the Limat model[12] for the symmetric ($\gamma_{SV} = \gamma_{SL}$) and asymmetric cases ($\gamma_{SV} \neq \gamma_{SL}$). The cusps in our data (blue and red circles in Fig. 2b) are highly bent toward the vapor side in case of water drops. As a result, the slope θ_{SV} (θ_{SL}), measured in our data as 100.10º (40.90º), is larger (smaller) than those calculated using simulation parameters based on the model (see Supplementary Table 2). Nevertheless, the asymmetric case is relatively well matched to our data with small deviations of $\Delta\theta_{SX}$ (= θ_{SX} (model) − θ_{SX} (exp.); –6.74º or 8.11º). In particular, we note that the solid angle calculated from the asymmetric case, $\theta_S = 37.62°$, is close to our experimental data, $\theta_S = 39.00°$. For EG 40% drops, the slopes are reasonably close to our experimental data in both cases, especially closer in the asymmetric case. These results indicate that the asymmetric case works better.
